# Anterior Skull Base Reconstruction in Multiportal Approaches: Insight into Vascularized Flap Techniques

**DOI:** 10.3390/jcm13237229

**Published:** 2024-11-28

**Authors:** Luca Ferlendis, Bianca Bossi, Antonio Tabano, Lidia Bifone, Alberto Daniele Arosio, Paula Nathalie Espinoza Apolo, Fabio Pozzi, Elisa Coden, Maurizio Bignami, Paolo Castelnuovo, Davide Locatelli

**Affiliations:** 1 Division of Neurosurgery, Department of Biotechnology and Life Sciences, University of Insubria, Ospedale di Circolo e Fondazione Macchi, Viale Luigi Borri, 57, 21100 Varese, Italy; 2 Division of Otorhinolaryngology, Department of Biotechnology and Life Sciences, University of Insubria, Ospedale di Circolo e Fondazione Macchi, Viale Luigi Borri, 57, 21100 Varese, Italy

**Keywords:** anterior skull base, multiportal, pericranial flap, nasoseptal flap, meningioma, sinonasal malignancies, cerebrospinal fluid leak

## Abstract

**Background/Objectives**: To evaluate the outcomes of anterior skull base (ASB) reconstruction using single versus double vascularized flap techniques following multiportal cranio-endoscopic approaches (CEA), based on a 12-year experience. **Methods**: A retrospective analysis was conducted on 46 patients who underwent ASB reconstruction after a CEA at our department between 2010 and 2022. Patients were divided into two groups: Group 1 received a pericranial flap (PF) reinforced with a fascia graft, while Group 2 underwent multiple flap reconstruction with PF, fascia graft, and nasoseptal flap (NSF). The primary outcome measured was the incidence of cerebrospinal fluid (CSF) leakage and the impact of adjuvant radiotherapy (RT) on reconstruction. **Results**: Group 1 (86.9%) demonstrated no significant postoperative CSF leaks, showing that the PF, combined with multilayer techniques (including underlay sealing matrix and overlay fascia graft), effectively repaired ASB defects. Group 2 (13.1%), employing both PF and NSF, showed similar outcomes, with the dual flap approach particularly beneficial in cases of post-traumatic fistulas or when the nasal septum was spared by disease. No significant differences were observed in complications or flap necrosis, even in patients receiving adjuvant RT. **Conclusions**: The PF is a reliable and versatile option for ASB reconstruction, often sufficient as a single-flap technique. The addition of an NSF can be beneficial in specific cases, particularly in post-traumatic conditions or tumors with unilateral endonasal invasion. However, PF alone, when combined with a multilayer approach, minimizes the risk of CSF leakage and long-term flap necrosis, underscoring the importance of tailored surgical strategies for optimal outcomes.

## 1. Introduction

Multiportal combined endoscopic endonasal and open transcranial approaches have gained popularity for the treatment of large tumors that extend into both the extra- and intracranial space [1,2,3,4]. Specifically, for ASB tumors or sinonasal malignancies invading the anterior cranial base with intradural invasion, an extended endonasal approach (eEEA) can be combined with an open transcranial approach [3,4].

A critical aspect of this surgical strategy is reconstructing the ASB, especially in the presence of large defects. A watertight dural seal provides a barrier between the paranasal sinuses and the intracranial space, maintaining a functional sinonasal system and optimizing aesthetic outcomes [2,5]. Failure to provide adequate reconstruction of skull base defects can result in significant complications, including CSF fistula formation, meningitis, and tension pneumocephalus [2,6,7,8]. Reconstruction with vascularized flaps is preferred over free grafts, especially when wound healing may be compromised by adjuvant or neoadjuvant radiation therapy (RT) or chemotherapy (CHT) [1]. Repair methods using vascularized flaps for the prevention of postoperative CSF leakage are popular [9,10], as they provide a robust, vascularized tissue for the closure of ventral ASB defects following endoscopic endonasal skull base tumor resection [2].

The pericranial flap (PF) is the workhorse for repairing ASB defects after bifrontal transcranial resection due to its rich blood supply and ease of harvesting [2,11,12]. The simultaneous use of the PF and nasoseptal flap (NSF) has proven effective in multiportal surgeries, offering extensive vascularized tissue coverage, which is critical for minimizing CSF leaks and ensuring good healing outcomes [1,2,13,14]. However, when addressing tumors invading the ASB, the use of the NSF is often infeasible. This is because the nasal septum is commonly affected by the disease, particularly in malignant lesions, necessitating the removal of its posterior two-thirds, which prevents the use of a vascularized NSF [14]. This constraint underscores the importance of alternative reconstructive procedures and the need for a flexible approach tailored to each patient’s unique anatomical and pathological challenges [2].

This article presents a twelve-year experience from our skull base unit in ASB reconstruction following multiportal cranio-endoscopic approaches (CEA), combining transcranial and endonasal techniques. It examines the reconstructive methods employed, along with anatomical considerations, surgical planning, and key intraoperative strategies for successful reconstruction. The aim is to evaluate the outcomes of ASB reconstruction using single (e.g., PF) versus double vascularized flap techniques (e.g., PF plus NSF) following CEA.

## 2. Materials and Methods

We conducted a retrospective review of all patients who underwent a combined endoscopic transnasal and transcranial approach (cranio-endoscopic approach (CEA)), followed by ASB reconstruction at our Skull Base Unit (University of Insubria, Ospedale di Circolo e Fondazione Macchi) between 2010 and 2022. Informed consent was obtained from all individual participants included in the study. For each patient, we collected data on demographics, pathology, previous surgeries, prior radiotherapy RT and CHT, surgical indication, ASB reconstruction method, intraoperative and postoperative complications, and follow-up outcomes. In oncological cases, we also evaluated additional factors such as the extent of resection (EoR), the use of adjuvant RT/CHT, and tumor recurrence.

**Figure 1:**
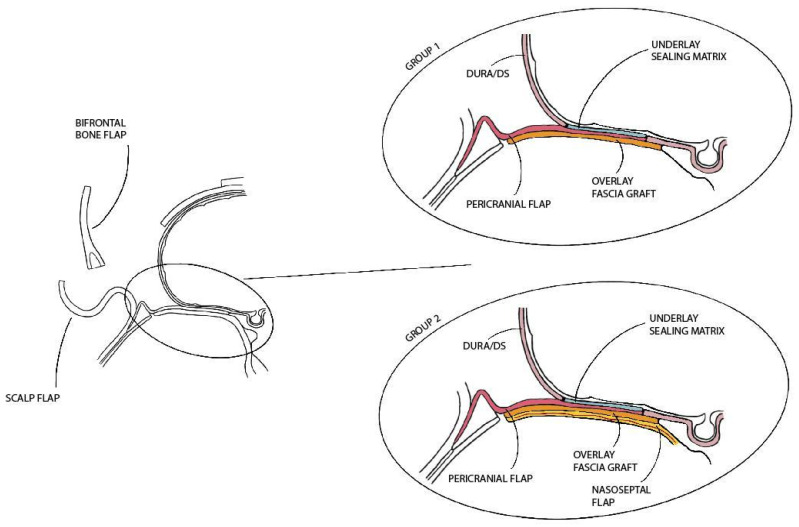
Schematic representation of the anterior skull base reconstruction methods used in Group 1 and Group 2. *Abbreviations*. DS: dural substitute.

Two study groups were defined based on the method of anterior skull base reconstruction (Figure 1).

- *Group 1* received a PF, reinforced with an underlay hemostatic sealing matrix and an overlay graft of fascia (either fascia lata or temporalis fascia);
- *Group 2* underwent a double flap reconstruction using both the PF and the NSF, with an intermediate layer of fascia graft for additional reinforcement.

The surgical technique is further detailed in Section 2.2.4.

The primary outcome assessed was the overall rate of post-operative CSF leak, as an indicator of ASB reconstruction failure.

### 2.1. Preoperative Evaluation, Surgical Indication and Exclusion Criteria

**Figure 2:**
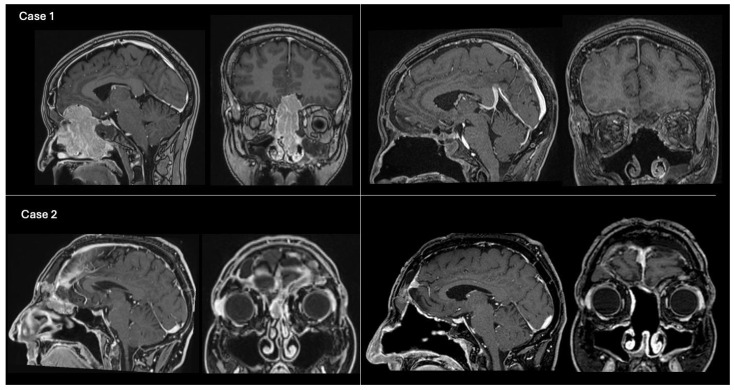
Example cases of preoperative and postoperative T1-weighted contrast-enhanced brain MRI. Case 1 (**above**): On the left, preoperative MRI on sagittal and coronal views showing an olfactory neuroblastoma (grade C, modified Kadish classification [15]) invading the anterior cranial fossa. The ASB was reconstructed using method 1 (pericranial flap + overlay fascia lata graft). The NSF was not available due to tumor invasion of the nasal septum mucosa. On the right, 1-month postoperative MRI showing gross total resection of the tumor. Case 2 (**below**): On the left, preoperative MRI on sagittal and coronal views depicting a recurrent ASB atypical meningioma (Grade 2, WHO 2021), previously operated via open approach, with unilateral extension into the right nasal fossa. The ASB was reconstructed using method 2 (pericranial flap + fascia lata graft + NSF). On the right, 2-year postoperative MRI in sagittal and coronal views, demonstrating total tumor removal and intact ASB reconstruction following adjuvant RT.

All patients underwent preoperative brain computed tomography (CT) scan and contrast-enhanced magnetic resonance imaging (ce-MRI). The indications for a CEA were as follows: intracranial tumors of the ASB infiltrating the nasal fossae, paranasal sinuses and orbits; sinonasal malignancies filling the frontal sinus or invading the ASB with intradural extension over the orbital roof and/or frontal lobes invasion (pT4a-T4b, N0, M0) [13] (Figure 2); post-traumatic ASB fractures with CSF leak; and post-surgical encephalocele. Exclusion criteria were pure endoscopic endonasal approaches or transcranial approaches.

### 2.2. Surgical Technique

#### 2.2.1. Operative Setup

The combined cranio-endoscopic approach involves a multidisciplinary team composed of neurosurgeons and ENT surgeons. The endoscopic endonasal and transcranial phases of the procedures are often performed simultaneously. The patient is placed in a supine position with the head elevated (anti-Trendelenburg position) and slightly flexed, placed over a gel head support. Standard equipment includes a neuronavigation system, 0- and 45-degree nasal endoscopes (Karl Storz, Tuttingen, Germany), an endoscopic endonasal set, straight and curved endoscopic instruments, and a microsurgical set. The transcranial approach can be performed with the assistance of either a microscope or an exoscope.

#### 2.2.2. Endoscopic Endonasal Phase

The surgical technique may vary according to the nature and extent of the anthology to be treated. The eEEA is performed using a two nostril–four hand technique for better exposure and instrument mobility by debulking the lesion to identify its origin and define its boundaries while preserving key anatomical landmarks. The posterior two-thirds of the nasal septum are removed using the two nostril–four hand technique for better exposure and instrument mobility [16]. This includes removing the rostrum sphenoidale to access the posteroinferior dissection margin [13]. Frontal sinus access is achieved with a Draf type III median sinusotomy if both sides are affected. When the nasal mucosa is invaded by the tumor, the septal branches of the sphenopalatine arteries are identified and coagulated to reduce bleeding and enhance visibility. If the nasal mucosa is invaded, septal branches of the sphenopalatine arteries are coagulated to reduce bleeding; otherwise, vascularization is preserved for potential use of an NSF [11].

After the exposition of posteroinferior and anterosuperior margins, a subperiosteal dissection of the naso-ethmoidal–sphenoidal complex follows, pushing it centrally using a centripetal technique. Depending on tumor extension, this step may be combined with a Type IIIa medial maxillectomy, achieved by removing the lacrimal bone and transecting the nasolacrimal duct a few millimeters distal to the lacrimal sac [16]. This maneuver optimizes surgical access to the maxillary sinus and is particularly effective for controlling the retro-lacrimal recess, a key area for achieving radical resection in oncological cases [4,16].

The lamina papyracea is identified as the lateral limit and then mobilized. Frozen sections are used to confirm clear margins, and further dissection continues as needed. If the anterior skull base is eroded, communication between the intracranial and endonasal spaces is addressed.

#### 2.2.3. Transcranial Phase

The PF is harvested before performing a bifrontal craniotomy, which is performed just above the orbital roof to minimize brain retraction and prevent constriction of the flap during ASB reconstruction [4]. After bone removal and bleeding control, the frontal sinus (FS) is cranialized by completely removing its mucosa and drilling down its posterior wall, integrating the sinus cavity into the ASB. Cranialization requires obliterating the nasofrontal duct to prevent potential pathways for mucus drainage or infection, reducing the risk of mucocele formation and chronic sinus infections within the skull. Once the sinus cavity is incorporated into the cranial cavity, dural opening and tumor resection proceed [4].

The endonasal corridor allows precise excision of the ethmoidal and medial orbital wall components, while the subfrontal approach provides access to the anterior cranial base, improving control over neurovascular structures.

#### 2.2.4. Reconstruction Phase

**Figure 3:**
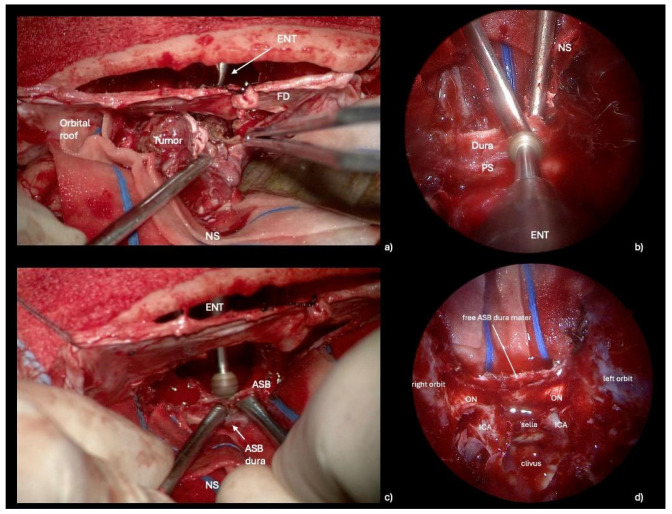
Intraoperative propedeutical steps to anterior cranial base reconstruction. (**a**) Microscopic transcranial view of simultaneous tumor removal: the ENT specialist and the neurosurgeon work together, utilizing multiportal access. (**b**,**c**) Endoscopic endonasal and microscopic transcranial views showing the crucial step of drilling the planum sphenoidale: by removing the bone, the dura mater of the anterior cranial fossa is exposed, facilitating its suturing to the pericranial flap during the reconstructive transcranial phase. (**d**) Final endoscopic view with the anterior cranial base dura mater exposed. *Abbreviations*. ENT: otolaryngologist; FD: frontal dura; NS: neurosurgeon; PS: planum sphenoidale; ASB: anterior skull base; ON: optic nerve; ICA: internal carotid artery.

**Figure 4:**
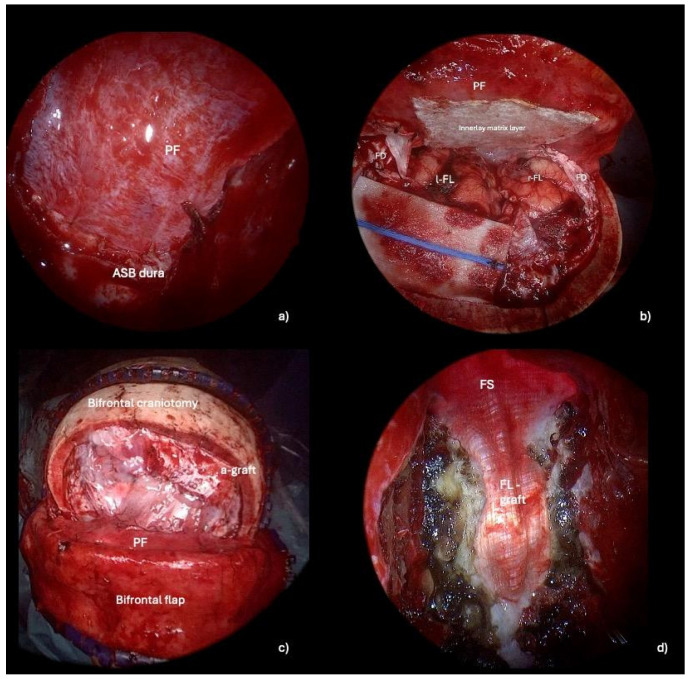
Intraoperative steps of ASB reconstruction method 1: pericranial flap plus autologous fascia lata overlay graft. (**a**) Endoscope-assisted suturing of the pericranial flap to the dura mater of the planum sphenoidale. (**b**) Transcranial view of the sutured PF with an underlay (intradural) hemostatic sealing matrix to stabilize the suture between the pericranial flap and the dura mater of the anterior cranial base. (**c**) Final transcranial view showing the frontal duraplasty made with an autologous pericranium graft and sutured with the autologous dura mater. (**d**) Final endoscopic endonasal view of the ASB reconstruction: the overlay fascia lata graft is stabilized with absorbable hemostatic material and fibrin glue. *Abbreviations.* PF: pericranium flap; ASB: anterior skull base; l-FL: left frontal lobe; r-FL: right frontal lobe; FD: frontal dura; a-graft: autologous graft; FS: frontal sinus; FL-graft: fascia lata graft.

The key to performing an optimal skull base reconstruction is to properly dissect the epidural space over the orbital roof laterally and the ethmoidal roof/planum sphenoidale posteriorly. Specifically, we recommend lightly drilling the lateral and posterior edges of the craniectomy via the endonasal corridor. Simultaneously, under microscopic view, the neurosurgeon dissects the epidural space while protecting the dura of the ASB, assisting the ENT specialist during this delicate phase. By removing the bone, the dura mater of the anterior cranial fossa is exposed, facilitating its suturing to the PF during the transcranial reconstruction. (Figure 3, Video S1). A transcranial approach is employed to repair the anterior skull base defect with a PF secured using button sutures. A hemostatic sealing matrix (e.g., TachoSil^®^) can be placed underlay (intradural) to stabilize the suture between the PF and the dura mater of the anterior cranial base. Concurrently, endoscope-assisted ASB reconstruction is performed. Additionally, connective tissue, such as temporal fascia or fascia lata, is applied in an overlay fashion to reinforce the reconstruction endonasally. Specifically, the fascia lata is carefully positioned as an overlay graft, covering the defect area to reinforce the primary reconstruction performed with the PF. (Figure 4). The surgical procedure is shown in Video S1.

When the nasal septum is unaffected by the tumor or in cases of post-traumatic defects, the septal branches of the sphenopalatine artery (SPA) can be preserved, allowing for the harvest of an NSF. This flap can then be used as a third layer in ASB reconstruction, providing robust vascularized tissue that enhances the structural integrity of the repair and promotes improved healing. Nasal packing is then applied and removed after 48 h. The bone flap is reattached using titanium plates and screws, while the skin flap is secured with button sutures.

### 2.3. Postoperative Management and Follow-Up

All patients underwent a brain CT scan on the first postoperative day to rule out complications. Intravenous third-generation cephalosporin therapy began the day prior to surgery and was continued for a minimum of 5 days. Patients remained on complete bed rest, positioned at a 30-degree incline, until the second postoperative day. Nasal packing was carefully removed within 48 h. Daily endoscopic medication was performed until discharge. Patients were advised to perform nasal irrigation with saline solution and apply mupirocin ointment twice daily for at least one month. Post-discharge follow-up included endoscopic endonasal examinations at 20, 40, 60, and 120 days after surgery. Neuroimaging follow-up was tailored according to the histopathological findings, with the first follow-up brain MRI generally performed at 1–3 months. The need for adjuvant RT/CHT was evaluated by a multidisciplinary team based on the tumor type.

## 3. Results

A total of 46 patients who underwent CEA followed by ASB reconstruction were included in this study. The mean patient age at the time of surgery was 58.6 years (range 32–84 years), with 35 patients (75%) being males and 11 (25%) females.

The demographic and clinical characteristics of the patients are summarized in Table 1. The patients included were further divided into three subgroups based on their pathology:

(1) S(1): oncologic cases (42 patients, 91.3%)
(2) S(2): post-iatrogenic fistulas following a pure eEEA (2 patients, 4.3%)
(3) S(3): post-traumatic fistulas (2 patients, 4.3%).

Among S(1), the most common presenting symptoms were nasal obstruction and hyposmia, reported by 23 patients, while 9 patients experienced visual disturbances such as diplopia and/or hypovision. Seven patients presented with epistaxis, and an additional seven had associated headaches. Tumor relapse was detected during follow-up in two asymptomatic patients.

Seven patients had received prior surgical treatment followed by adjuvant RT, and of these, two had also undergone chemotherapy (CHT). Histopathological analysis revealed 11 meningiomas (4 of which were atypical, WHO grade II), 9 sinonasal squamous cell carcinomas (SCC), 9 sinonasal intestinal-type adenocarcinomas (ITAC), and 4 esthesioneuroblastoma (ENB). Gross total resection (GTR) was achieved in 30 cases (65%), while subtotal resection (STR) was performed in 12 patients (34%).

Two cases involved delayed iatrogenic fistulas—S(2) following pure eEEA—with one case complicated by a postsurgical encephalocele.

In S(3), one patient presented with fever and the other with rhinoliquorrhea as the primary symptom.

Regarding anterior skull base reconstruction (Figure 1), Group 1 consisted of 40 patients (86.9%), including all cases of S(2) and 38 oncologic cases S(1). Group 2 included 6 patients (13.1%), comprising the S(3) cases and 4 oncologic cases S(1): specifically, 3 cases of ASB meningiomas with unilateral nasal extension and 1 case of unilateral spinocellular carcinoma (SCC).

Frontal duraplasty was performed in all cases, utilizing autologous dura when available in 35 cases, with reinforcement using autologous graft, pericranium, or fascia in 3 of these cases. On the other hand, a synthetic dural substitute (DS) was used in 11 cases.

Neither Group 1 nor Group 2 reported any cases of CSF leakage.

Postoperative complications included one case of meningitis, one case of rhinosinusitis, intracranial hemorrhage in two patients, and hydrocephalus in two patients (one postoperative and one post-traumatic). All of these occurred in S(1).

In S(1), 23 patients received adjuvant treatment: 18 with radiotherapy (RT) and 5 with RT plus chemotherapy (CHT). The total mean follow-up period was 60 months (range: 12–144 months), with evidence of recurrence in 14 cases within S(1). Of these 14 cases, only 4 underwent reoperation, all via a transcranial approach. These four cases comprised specifically of one case of ameloblastoma, one case of grade 2 meningioma, one case of grade 3 SCC, and one case of ENB. In these cases, the ASB was neither altered nor modified.

During the follow-up, particularly after RT, no cases of necrosis of the anterior skull base reconstruction flaps were observed. The operative and postoperative data are summarized in Table 2.

## 4. Discussion

Cranioendoscopic resection of tumors involving or originating from the ASB may result in large defects. Reconstructing these defects presents a unique surgical challenge, and the literature reports various reconstructive techniques. The simultaneous nature of the multiportal approach offers crucial technological and surgical advantages, enhancing the precision and effectiveness of these complex procedures and reducing frontal lobe retraction [4,17,18].

However, inadequate ASB reconstruction can lead to postoperative CSF leakage, potentially resulting in life-threatening conditions such as meningitis and tension pneumocephalus. Ngo et al. [19] classify defects larger than 2 cm as large, while Yano et al. [20] have proposed anatomical classification systems for ASB defects to aid in selecting the appropriate surgical reconstruction techniques, with a focus on flap selection. Additionally, Ryan et al. [21] have proposed an algorithmic approach to select the appropriate surgical strategy, recommending the use of a regional flap, such as a PF, for large defects. Vascularized flaps are particularly effective when wound healing may be compromised by adjuvant radiotherapy [2].

Some authors [1,2,13,14] have reported the simultaneous use of two vascularized flaps, such as the PF and the NSF, in the context of tumor-related pathologies. They suggest that this combined approach leads to better outcomes compared with the sole use of PF. Postoperative RT may compromise the vascularization of the PF, which can result in flap necrosis and subsequent delayed CSF leakage [2,22]. Consequently, these authors argue that the use of two vascularized flaps may provide a more secure reconstruction of the ASB, addressing issues associated with using the PF alone.

Nevertheless, our data analysis has revealed that a double-flap technique is not feasible in most oncologic cases [S(1)] due to involvement of the nasal septum by the disease. To increase the EoR and achieve a GTR, removal of the posterior two-thirds of the nasal septum is often necessary, which frequently precludes the use of a vascularized NSF [14]. Building on over a decade of experience, we conducted a retrospective analysis to evaluate outcomes across various reconstruction techniques for large ASB defects. Our 12-year, single-center experience provides two significant advantages compared with previous studies [1,2,13,14]: a larger patient cohort and an extended follow-up period, allowing for a more comprehensive assessment of long-term outcomes.

We identified two groups based on reconstruction techniques: in Group 1 (86.9%) we did not use the NSF due to the necessity of sacrificing the SPA branches during endoscopic resection, while in Group 2 we utilized a multilayer double flap technique using both the PF and the NSF.

Our study included multiple cohorts [S(1), S(2) and S(3)] to reflect the clinical conditions that influence the feasibility of a double-flap reconstruction technique (Group 2) versus a single-flap approach (Group 1).

The primary outcome assessed was the overall rate of post-operative CSF leaks, serving as an indicator of ASB reconstruction failure. Notably, no cases of CSF leakage were reported in either Group 1 or Group 2, even after RT in oncologic cases. This finding underscores the importance of selecting the appropriate reconstructive technique based on the underlying pathology, optimizing the surgery’s purpose, whether that be to maximize the EoR in oncologic cases or to repair the fistula in the other groups.

Specifically, double-flap reconstruction with an additional NSF is feasible mainly in cases of unilateral oncologic involvement or in cases within S(3), where the NSF can be isolated and preserved. In contrast, in oncologic cases with bilateral extension [S(1) and S(2)], SPA sacrifice is often necessary, which precludes NSF harvesting. In particular, for cases in S(2), the NSF was sacrificed during the initial surgery. Consequently, only a single-flap technique (Group 1) can be applied in these scenarios.

The NSF is used when there is no risk of oncological tissue dissemination and the necessity to sacrifice the NSF in extensive oncologic resections underscores the need for alternative multilayered techniques to provide robust sealing in ASB reconstructions. In our experience, while it is beneficial to add the NSF when available, its use does not significantly impact the outcome of ASB reconstruction performed with a single-flap technique. In fact, Group 1 represented 86.9% and only the PF was used as a vascularized flap, and the short- and long-term results were found to show no significant complications. However, according to us, the surgical technique should be performed in a multilayer fashion: watertight suturing of the PF to the ASB dura mater under endoscopic assistance, the application of an absorbable intradural sealing matrix (e.g., TachoSil^®^), and an endonasal overlay graft of temporal fascia or fascia lata. This additional layer helps to provide structural support and prevent CSF leakage, ensuring a robust and effective reconstruction. The dural exposure ensured by minimal drilling of the inferior and lateral margins of the craniectomy is crucial for facilitating the suturing of the PF to the dura of the anterior skull base. Moreover, the multiportal approach allows for dual-point assistance and should be leveraged by performing lavage tests via the transcranial route under endoscopic endonasal guidance. Our findings validate the effectiveness of a single-flap approach in scenarios where NSF use is precluded, demonstrating that the PF technique alone can maintain structural integrity and prevent CSF leakage even in complex ASB reconstructions.

On the other hand, the use of two flaps for reconstruction is associated with reduced postoperative nasal crusting as the NSF likely promotes better and faster nasal mucosal regeneration [14,23]. This reduction in crust formation can lead to a faster and more efficient healing process of the endonasal surgical site. In contrast, healing of the fascia used in reconstruction usually requires several months to fully cicatrize, as avascular tissues typically take longer to integrate and regenerate when compared with vascularized flaps [14]. Consequently, the patients with fewer nasal crusts require less frequent saline irrigations, leading to greater comfort and a reduction in the postoperative discomfort associated with nasal cleaning. A recent systematic review [24], however, found no significant differences in quality of life (QoL) outcomes associated with the use of an NSF after endoscopic skull base surgery. Nonetheless, it is important for patients to be informed that extensive surgical approaches and the use of an NSF can adversely impact QoL due to sinonasal symptoms [24].

The importance of vascularized flaps in ensuring successful healing after ASB expanded approaches is evident, with the double-flap technique proving more effective when feasible. Our findings do not reveal a direct link between RT and ASB reconstruction failures, even as we managed progressively larger and more complex lesions over time. While RT did not significantly impact reconstruction outcomes in our cohort, it remains a critical factor when selecting the most suitable reconstructive technique. We generally avoided bony grafts and synthetic materials due to their heightened risk of infection and extrusion, making vascularized pedicled flaps the preferred option whenever possible.

Managing surgical failure requires prompt and careful intervention. While we do not routinely use lumbar drains (LD), they may be considered for conservatively managing low-to-moderate postoperative CSF leaks [25]. However, if postoperative imaging reveals progressive pneumocephalus, LD should be avoided and immediate revision surgery is necessary. Similarly, early surgical revision is strongly recommended in cases of significant CSF leakage [25].

### Limitations

The main limitation of this study is the imbalance between the two patient cohorts, with most cases falling into the group where the NSF could not be utilized due to extensive tumor resection. This reflects the inherent challenge in achieving GTR when oncological involvement of the nasal septum necessitates its removal.

Another limitation is the presence of the mixed cohort, comprising oncologic cases, post-iatrogenic fistulas, and post-traumatic fistulas. However, these different cohorts were intentionally analyzed to reflect the clinical reality in which ASB reconstruction techniques are applied across various indications, highlighting the versatility and robustness of the methods under different pathological conditions.

Additionally, the retrospective observational design may introduce selection bias and limits control over confounding factors. Furthermore, no statistical analysis was performed due to the retrospective and descriptive nature of this study and the inherent non-comparability of cases in Groups 1 and 2. Potential variations in surgical expertise over the 12-year study period may have also influenced outcomes. Lastly, while the follow-up period is substantial, it varies across patients, complicating the consistent assessment of long-term reconstruction durability. Future studies with larger, more homogenous cohorts could permit a more rigorous statistical evaluation of these techniques.

## 5. Conclusions

Our twelve-year experience with ASB reconstruction utilizing multiportal CEA demonstrates that a single-flap technique with a PF alone is highly efficient for sealing large defects, often without the need for NSF. While combining PF with NSF can be advantageous, particularly in post-traumatic cases or unilateral tumor expansions, PF alone, when applied with a multilayer reconstructive technique, produces reliable results. This approach, using watertight suturing, underlay absorbable hemostatic agents, and overlay grafts, effectively prevents CSF leaks and ensures long-term structural integrity. This study underscores the adaptability required in ASB reconstruction, and supports a tailored, pathology-driven approach to maximize surgical success and minimize patient morbidity.

## Figures and Tables

**Table 1:** Patient demographics and clinical presentation.

Patient Demographics and Clinical Presentation		
**Mean age at surgery**		59
**Gender**		
	Female	11
	Male	35
**Presenting signs and symptoms**		
	Nasal obstruction and hyposmia	23
	Visual disturbances	9
	Asymptomatic (in FU)	2
	Epistaxis	7
	Headache	7
	Others (fever, oral mass, seizures)	5
**Pathology**		
**(1) Oncologic cases S(1)**		
	Common meningioma (WHO grade I)	7
	Atypical meningioma (WHO grade II)	4
	Sinonasal inverted papilloma	1
	SCC	9
	ITAC	9
	ENB	4
	SNEC	1
	Bone dysplasia	1
	MPNST	1
	Biphenotypic sinonasal sarcoma	1
	Chondrosarcoma	1
	Giant cell tumor	1
	Frontal osteoma	1
	Ameloblastoma	1
**(2) Iatrogenic fistula after eEEA S(2)**		
	Iatrogenic fistula	2
**(3) Post-traumatic fistula S(3)**		
	Post-traumatic fistula	2
**Previous Treatment (Surgery + RT/CHT)**		7

FU: follow-up; SCC: sinonasal squamous cellular carcinoma; ITAC: sinonasal intestinal type adenocarcinoma; ENB: esthesioneuroblastoma; MPNST: malignant peripheral nerve sheath tumor; SNEC: small cell neuroendocrine carcinoma; RT: radiotherapy; CHT: chemotherapy.

**Table 2:** Operative and postoperative summary.

Operative and Postoperative Summary		
**ASB reconstruction**		
	PF + overlay graft (fascia lata or temporalis muscle fascia)	40
	PF + overlay graft + NSF	6
**Complications**		
	Meningitis	1
	Rhinosinusitis	1
	Intracranial hemorrhage	2
	Hydochepalus	2
**Type of tumor resection**		
	Gross total resection	30
	Subtotal resection	16
**Adjuvant therapy**		
	RT	18
	RT + CHT	5
**Follow up (mean)**		60 months
	Recurrence	14
	N/A	5

ASB: anterior skull base; PF: pericranial flap; NSF: nasoseptal flap; RT: radiotherapy, CHT: chemotherapy.

## Data Availability

The original contributions presented in the study are included in the article/supplementary material, further inquiries can be directed to the corresponding author/s.

## Supplementary Materials

The following supporting information can be downloaded at https://www.mdpi.com/article/10.3390/jcm13237229/s1, Video S1: Anterior skull base reconstruction in multiportal cranioendoscopic approach.
